# Targeting the NLRP3–NEK7 axis with rebamipide: Drug repurposing in Parkinson’s disease

**DOI:** 10.4103/NRR.NRR-D-25-01189

**Published:** 2025-12-30

**Authors:** Hye-Sun Lim, Gunhyuk Park

**Affiliations:** Herbal Medicine Resources Research Center, Korea Institute of Oriental Medicine, Naju-si, Jeollanam-do, Republic of Korea

**Introduction — from traditional wisdom to modern challenges:** Drug repurposing has become a strategic pillar in pharmaceutical development, addressing the rising challenges of novel drug discovery (Saranraj and Kiran, 2025). The conventional pipeline is often arduous > $2 billion per approval, with < 10% clinical success. The burden is even heavier in programs in the central nervous system (CNS) due to the blood–brain barrier, disease heterogeneity, and limited biomarkers (Saranraj and Kiran, 2025). These inefficiencies create a bottleneck for neurodegenerative diseases, underscoring the need for faster, lower-risk strategies such as drug repurposing to deliver therapies more quickly (Saranraj and Kiran, 2025).

Parkinson’s disease (PD) exemplifies this pressing need. It is the world’s fastest-growing neurodegenerative condition, affecting 11.8 million people globally and imposing an annual economic burden exceeding $52 billion in the United States alone in 2025, projected to reach over $79 billion by 2037 (Henriquez and Narayan, 2023). The global PD-therapeutics market is valued at approximately $5.85 billion in 2025, with projections indicating growth to $9.39 billion by 2032, driven by increasing patient numbers and ongoing research (Henriquez and Narayan, 2023). While symptomatic relief is available, the pursuit of disease-modifying therapies remains a critical unmet need, with over sixty active clinical studies underway. This immense societal and economic burden directly propels a shift in drug-development paradigms, fostering collaborative approaches between industry, research-communities, and patient-advocacy groups (Henriquez and Narayan, 2023).

In this landscape, drug repurposing, which involves identifying new therapeutic indications for existing or previously researched drugs, presents a highly rational and attractive strategy. It offers substantial advantages, including reduced development time, lower costs, and significantly de-risked pathways due to established safety profiles and pharmacokinetic data (Saranraj and Kiran, 2025). This approach fundamentally transforms the probability of success by mitigating the most common and costly causes of drug-development failure: unexpected toxicity and unfavorable pharmacokinetics (Saranraj and Kiran, 2025). By leveraging drugs with known human safety and predictable CNS penetration, repurposing bypasses formidable early-stage hurdles, making it particularly appealing for complex neurological disorders.

**Figure 1:**
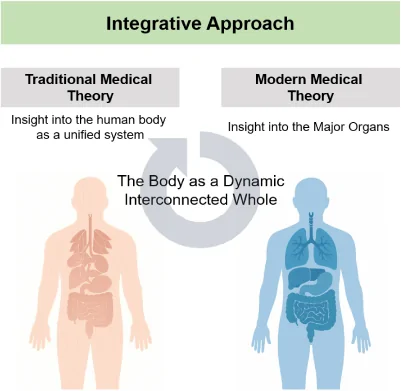
Integrative approach to understanding the human body. This figure illustrates an integrative approach to understanding the human body by combining Traditional Medical Theory and Modern Medical Theory. The left side represents the Traditional Medical Theory, emphasizing the interconnectedness of body systems, while the right side represents the Modern Medical Theory, focusing on organ systems. The central concept highlights the dynamic interaction between all parts of the body, emphasizing the body as a unified whole. Created with assistance from ChatGPT.

By re-examining ancient wisdom through the lens of modern pharmacology, we can uncover novel therapeutic applications (**Figure 1**). This study demonstrates this by exploring rebamipide (Mucosta®), a gastric muco-protective agent, and its potential to modulate the NOD-, LRR- and pyrin domain-containing protein 3–NIMA related kinase 7 (NLRP3–NEK7) axis for PD treatment, bridging traditional medical theory with contemporary pharmacological evidence.

**Rebamipide’s novel potential — bridging traditional insight and molecular mechanisms: *Rebamipide’s established profile and expanding pharmacological actions:*** Rebamipide is a quinolone derivative widely utilized in Asia as a gastric muco-protective agent for conditions such as gastritis, ulcers, and mucosal damage (Kak, 2025). Its mechanism involves stimulating prostaglandin synthesis, increasing gastric mucosal blood flow, promoting mucus secretion, and enhancing mucosal defense (Kak, 2025). Beyond its gastrointestinal applications, rebamipide exhibits free-radical-scavenging and anti-inflammatory effects (Kim et al., 2013; Kak, 2025). Its extensive clinical use and favorable safety profile, with few and mild adverse drug reactions, make it an exceptionally strong candidate for drug repurposing. In 2020, the outpatient prescription market of rebamipide in Korea alone reached $90 million, underscoring its widespread acceptance and established market presence (Kak, 2025). This proven safety and tolerability in millions of patients can significantly shorten the most costly and time-consuming stages of drug development (Phase 1/2 safety), rendering the repurposing process highly efficient and economically attractive (Kak, 2025).

Emerging research indicates that the therapeutic scope of rebamipide extends beyond the gastrointestinal tract to systemic immunomodulatory functions (Kak, 2025). Its anti-inflammatory properties have been demonstrated in diverse conditions, including dry-eye disease, bladder inflammation, and atopy, primarily through the suppression of nuclear factor-κB signaling (Qiao et al., 2022). Furthermore, rebamipide has shown neuroprotective properties in neurological disorders, reducing Aβ production and improving cell viability in Alzheimer’s models, and mitigating PD through mitochondrial bioenergetic stabilization and enhanced antioxidant activity (Fukui et al., 2017; Lim et al., 2025). Building on these observations, a mechanistic bridge to PD emerges: beyond gastroprotection, rebamipide’s stimulation of prostaglandin synthesis and scavenging of reactive oxygen species act as an upstream brake on neuroinflammatory cascades (Kak, 2025). Prostaglandin-mediated immunomodulation tempers excessive inflammatory signaling, while reactive oxygen species reduction stabilizes mitochondrial function in vulnerable dopaminergic neurons, diminishing redox-driven danger signals. Together, these actions operate upstream of inflammasome activation to attenuate the NLRP3–NEK7 axis and downstream interleukin (IL)-1β/IL-18 release, thereby reducing microglial priming and supporting dopaminergic neuron survival and motor outcomes.

**Traditional “Bi” concept and its connection to the gut–brain axis:** Eastern traditional medicine has historically emphasized the intricate interplay between internal organs, particularly highlighting the connection between digestive organs and mental health. Within this framework, “Bi” is a functional concept distinct from the anatomical spleen (Jeong and Kim, 2024). It holistically governs digestion, the transportation and transformation of nutrients, as well as cognitive and emotional functions, encapsulated by the expressions “Bi Zhu Yun Hua” (Bi governs transportation and transformation) and “Bi Zhu Yi” (Bi governs thought/intention) (Jeong and Kim, 2024). This traditional understanding provides a framework for explaining prodromal and clinical symptoms of PD, such as fatigue, depression, appetite loss, and cognitive decline, which often align with imbalances in “Bi” function. The “Bi” concept can be viewed as an ancient systems-biology concept encompassing integrated digestive, cognitive, and emotional functions (Zhao et al., 2024). This holistic perspective bears striking similarities to modern scientific understanding, such as the gut–brain axis, enabling novel pathophysiological interpretations of neurodegenerative diseases. It suggests that traditional medical principles can serve as a rich source of hypotheses for identifying systemic therapeutic targets for complex modern diseases such as PD (Aburto and Cryan, 2024). The gut–brain axis represents a bidirectional communication pathway through which gut inflammation and intestinal barrier dysfunction can influence brain function via neural, endocrine, and immune routes (Aburto and Cryan, 2024). In patients with PD, a consistent association between alterations in gut microbiota and neuropathology has been reported (Aburto and Cryan, 2024; Zhao et al., 2024). The actions of rebamipide in strengthening the intestinal barrier, suppressing inflammation, and modulating the microbiota align with mechanisms of systemic-inflammation alleviation and brain-inflammation suppression via this axis, making it a suitable candidate for a nervous system anti-inflammatory agent mediated by the gut–brain axis.

**Tissue distribution and pharmacokinetic considerations of rebamipide:** Radiolabeled studies indicate that rebamipide localizes predominantly to the gastrointestinal mucosa, with relatively high levels in the stomach and intestines and appreciable exposure in the liver and kidneys; brain penetration appears modest rather than absent, with emerging reports suggesting some central exposure (Fukui et al., 2017; Kak, 2025; Lim et al., 2025). Pharmacokinetically, rebamipide shows very high plasma protein binding (**~**98%), undergoes hepatic biotransformation mainly via CYP3A4 to 6- and 8-hydroxy metabolites, and has an elimination half-life of approximately 2 hours (Kak, 2025). Absorption is poorer in the acidic upper gastrointestinal tract and better distally, often producing biphasic (dual-peak) plasma profiles. In the context of PD, these properties support a dual-route rationale: an indirect pathway whereby gut–brain signaling and peripheral attenuation of the NLRP3–NEK7 inflammasome reduce IL-1β/IL-18 output and microglial priming; and a complementary, limited direct CNS component consistent with modest central exposure. Overall, peripheral and gut-derived immunomodulation likely constitutes the principal conduit to central anti-inflammatory and neuroprotective effects, with any direct CNS action serving as an adjunct mechanism.

**Figure 2:**
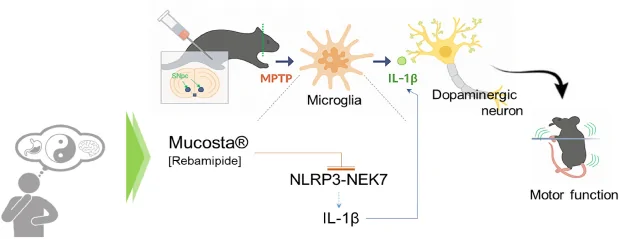
Proposed mechanism of neuroprotective effects of rebamipide in Parkinson’s disease. “Pi (spleen)” is introduced here solely as a traditional conceptual framework that inspired this mechanistic hypothesis and is not an anatomical designation. The figure illustrates only modern biological pathways and does not imply a direct causal relationship. Rebamipide (Mucosta®), a clinically approved drug, is proposed to exert protective effects against the pathogenesis of Parkinson’s disease by alleviating neuroinflammation and dopaminergic neurodegeneration through NLRP3 inflammasome inhibition. Upon the administration of MPTP, a neurotoxin that induces Parkinson’s-like symptoms in mice, microglia become activated, leading to the release of the pro-inflammatory cytokine IL-1β, which contributes to dopaminergic neuron degeneration. The action of rebamipide interferes with this cascade, ultimately protecting dopaminergic neurons and alleviating the associated motor dysfunction in the model, as seen by reduced tremor-like behaviors in the treated mice. Reprinted with permission from Lim et al. (2025). IL: Interleukin; MPTP: 1-methyl-4-phenyl-1,2,3,6 tetrahydropyridine; NEK7: NIMA related kinase 7; NLRP3: NOD-, LRR- and pyrin domain-containing protein 3.

**Molecular and experimental evidence for NLRP3-NEK7 axis targeting:** To elucidate the action of rebamipide on CNS inflammation, molecular dynamics-based simulations and pharmaco-net analysis were conducted. These analyses revealed that rebamipide possessed high-affinity binding sites on NLRP3, including the interface with NEK7, suggesting an allosteric inhibition of inflammasome assembly. Molecular-docking analysis showed that rebamipide exhibited the strongest binding affinity for the NLRP3-NEK7 complex (–10.5 kcal/mol for PDB: 6NPY), surpassing its affinity for NLRP3 alone or ASC. This indicates that rebamipide interacts through hydrogen bonds and halogen interactions with ASN978, TYR1009, and PRO1034 of NLRP3 and LYS163 of NEK7, implying a role in disrupting or stabilizing their interaction. Surface plasmon resonance analysis experimentally validated the direct binding of rebamipide to the NLRP3 protein, demonstrating notable kinetic affinity and supporting its role as a novel NLRP3 inflammasome inhibitor. Binding affinity of rebamipide with both NLRP3 and the NLRP3-NEK7 complex increased with its rising concentrations. This robust molecular-level validation of interaction strongly supports rebamipide as a rational and economically viable approach to targeting the NLRP3-NEK7 axis. In an MPTP (1-methyl-4-phenyl-1,2,3,6 tetrahydropyridine)-induced mouse model of PD, rebamipide effectively suppressed microglial activation and preserved dopaminergic neurons in the substantia nigra. Specifically, it significantly reduced levels of neuroinflammatory cytokines. Rebamipide treatment protected dopaminergic neurons and preserved dopamine levels, leading to the alleviation of motor dysfunctions, including bradykinesia. Crucially, the neuroprotective effects of rebamipide were not observed in *NLRP3*-gene knockout mice. This unequivocally demonstrates that the beneficial effects of rebamipide specifically depend on its ability to modulate the NLRP3 inflammasome pathway, firmly establishing a direct causal link between its mechanism of action and its therapeutic efficacy in PD models (Lim et al., 2025; **Figure 2**).

**Systemic inflammation and inter-organ interconnectivity:** The understanding of neuroinflammation is expanding beyond the CNS and gut-brain axis to encompass other systemic connections, underscoring that inflammation in peripheral organs can significantly impact brain health and neurodegenerative processes (Aburto and Cryan, 2024). This evolving paradigm recognizes the brain not as an isolated entity but as an integral part of a larger, interconnected system, wherein dysfunction in one organ can propagate inflammatory signals to the CNS (Aburto and Cryan, 2024). This highlights that neuroinflammation and neurodegeneration are not solely brain-confined phenomena but are profoundly influenced by systemic factors and the health of peripheral organs. This opens new avenues for therapeutic intervention by targeting systemic inflammation or peripheral-organ dysfunction to benefit the CNS. A growing body of research supports these inter-organ connections, demonstrating how various axes contribute to neuroinflammation. For instance, the gut–brain axis is a well-established example: Gut inflammation and intestinal barrier dysfunction can influence brain function via neural, endocrine, and immune routes (Aburto and Cryan, 2024). The theoretical basis underlying these modern inter-organ research findings bears striking similarities to the holistic concepts found in traditional medicine (**Figure 1**). Traditional medical systems often view the body as an integrated network wherein the health of one organ system profoundly impacts others, including the brain. This perspective, exemplified by the “Bi” concept’s broad influence on digestion, cognition, and emotion, aligns remarkably with the contemporary understanding of systemic inflammation and organ-brain axes. The ability of rebamipide to inhibit NLRP3 inflammasome activation suggests its potential to exert neuroprotective effects through these diverse organ-brain axes (Lim et al., 2025). The strategy of modulating “inflammasomes” as a common molecular node in inflammatory diseases could lead to universal therapies for various pathologies beyond PD (Lim et al., 2025). If the NLRP3 inflammasome acts as a shared pathway affecting multiple organ systems in various inflammatory conditions, a drug that effectively targets this node, such as rebamipide, then holds broad, “universal” therapeutic applicability. This implies that drug-repurposing potential of rebamipide could extend far beyond PD, positioning it as a highly valuable asset in the pharmaceutical landscape (Kak, 2025).

**Conclusion — a convergent strategy for future neurodegenerative therapies:** Drug repurposing is increasingly recognized as an economically viable and rational approach to developing treatments, particularly for complex neurological disorders. Its advantages, including reduced development time, lower costs, and de-risked development pathways, make it attractive in an era of escalating research and development expenses and high failure rates. Beyond its economic benefits, drug repurposing can be enhanced by integrating insights from traditional medicine. Traditional theories provide a unique framework for identifying new therapeutic applications, with their empirical knowledge and holistic concepts serving as innovative “discovery engines.” By systematically re-evaluating such insights through modern pharmacology and systems biology, researchers can uncover novel strategies that conventional screening might overlook. The rebamipide case exemplifies this convergence of traditional wisdom and modern validation. Its repurposing for PD, guided by the “Bi” concept and confirmed through NLRP3-NEK7 inhibition, offers a compelling model for drug development. This study clarifies the functional meaning of “Bi” and illustrates how traditional knowledge can inform modern pharmacology by repositioning existing agents into viable therapies. Three pillars support this strategy: (1) translating concepts such as “Bi” into modern physiology; (2) adopting a systemic view of disease to identify shared inflammatory targets such as NLRP3; and (3) enabling repurposing by applying safe, approved drugs to new indications. Amid aging populations and rising neurodegenerative diseases, integrating traditional wisdom with modern science offers a promising discovery paradigm exemplified by rebamipide with the potential to unlock new treatments and address urgent neurological needs.

*This work was supported by a grant on the development of sustainable application for standard herbal resources (KSN1823320), by a grant on the development of innovative technologies for the future value of herbal medicine resources (KSN2511030), and development of an upcycling platform technology for food waste utilization (KSN2511040) from the Korea Institute of Oriental Medicine, and by a grant on the Pharmacokinetic interaction and mechanistic investigation using isobologram and multi-omics analysis of traditional Korean medicine and western medicine concomitant therapy for establishing therapeutic basis in the treatment of Alzheimer’s disease (RS-2024–00352796) from the National Research Foundation of Korea, Republic of Korea (to GP).*
